# The Annual Meeting of the Pharmaceutical Society

**Published:** 1913-05-24

**Authors:** 


### The Annual Meeting of the Pharmaceutical Society.

The report of the Council which was presented at the seventy-second annual meeting of the Pharmaceutical Society on Wednesday at 17 Bloomsbury Square reveals quite a satisfactory state of affairs. The membership is 7,522, being larger than it has ever been, and representing an increase during the past ten years of 2,000. During the past year 874 candidates were examined for the qualification of chemist and druggist, and of this number 395 passed, and their names were added to the Register. The number of cases of alleged infringement of the Pharmacy Act which were investigated was 1,362, and in 238 cases legal proceedings were instituted. With regard to the Insurance Act the report states that the trial period of (the Insurance Act) three months has passed, and nearly all the pharmacists who, perhaps doubtfully, took service in January, have now agreed to continue for a further nine months to complete a year's experience of the working of the Act. In moving the adoption of the report the President referred to a Bill which has been drafted, providing for the establishment of a dispenser's qualification. He said it was not the intention of the Council to weaken in any way the status of the pharmacist, but to give statutory recognition to persons who had had three years' practical experience in dispensing prescriptions, and had passed an approved examination. A brief reference was recently made to the proposed new measure in The Hospital, and it is felt that some steps ought to be taken to ensure an adequate supply of examined dispensers to act as assistants to pharmacists on the Insurance panels.

